# Melioidosis, an emerging infectious disease in the Midwest Brazil: A case report

**DOI:** 10.1097/MD.0000000000015235

**Published:** 2019-04-19

**Authors:** Cláudia Elizabeth Volpe-Chaves, Ana Cláudia Souza Rodrigues, Mara Luci Gonçalves Galiz Lacerda, Caroline Tieppo Flores de Oliveira, Suse Barbosa Castilho, Caroline Franciscato, Ivson Cassiano de Oliveira Santos, Ana Paula D’alincourt Carvalho Assef, Leonardo Roever, Sandra Maria do Valle Leone de Oliveira, Anamaria Mello Miranda Paniago

**Affiliations:** a Graduate Program in Infectious and Parasitic Diseases of Federal University of Mato Grosso do Sul; b Graduate Program on Health and Development in West Central Region of Federal University of Mato Grosso do Sul; c UNIDERP – Medical School; d Regional Hospital of Mato Grosso do Sul, Campo Grande, Mato Grosso do Sul; e Hospital Infection Research Laboratory, Oswaldo Cruz Foundation, Rio de Janeiro, Rio de Janeiro; f Federal University of Uberlandia, Department of Clinical Research, Brazil.

**Keywords:** *Burkholderia pseudomallei*, melioidosis, *Pseudomonas pseudomallei*, sepsis

## Abstract

**Rationale:**

Melioidosis is an emerging infectious disease in Brazil and caused by *Burkholderia pseudomallei,* with high morbidity and mortality rates. A total of 28 melioidosis cases were reported in Brazil until 2015. The majority of melioidosis cases were reported in the Northwest region of Brazil and such cases were not previously detected in the Midwest region of Brazil.

**Patient concerns:**

A 42-year-old man was admitted with a non-productive cough, dyspnea, myalgia, diffuse abdominal pain. Pulmonary auscultation revealed a vesicular murmur, snoring sounds, and the presence of basal crackling rales in the left hemithorax. The patient evolved with several respiratory failures and he was diagnosed as the first case of community-acquired pneumonia with sepsis caused by *B pseudomallei* in Mato Grosso do Sul, Midwest state of Brazil.

**Diagnosis:**

The cell isolates were subjected to 16S rRNA gene sequencing to confirm the bacterial species.

**Interventions:**

Administration of trimethoprim/sulfamethoxazole and meropenem stabilized the clinical condition of the patient. Subsequently upon discharge, the patient was also treated with trimethoprim/sulfametothoxazole for a year.

**Outcome:**

We reported the first case of community-acquired pneumonia with sepsis caused by *B pseudomallei* in Mato Grosso do Sul, Midwest state of Brazil and the patient survived.

**Lessons:**

The emergence of melioidosis in the Midwest region is being neglected and underestimated and melioidosis must be considered of the differential diagnosis in community infections.

## 1 Introduction

Melioidosis is an emerging infectious disease in Brazil and caused by *Burkholderia pseudomallei* previously known as Whitmore bacillus, *Pseudomonas pseudomallei*, and *Malleomyces pseudomallei*.^[1,2]^ This disease is associated with high morbidity and mortality rates and reliable pathognomonic features remain unidentified.^[3]^

Melioidosis was initially described in the early 20th century among animals in Aruba^[4]^; in Brazil, the first human case was reported in 2003 at Tejuçuoca-Ceará, Northeast region of Brazil, where 3 out of 4 children from the same family died of multiple organ systems failures.^[5]^

The transmission of *B pseudomallei* is associated with inhalation, inoculation, or ingestion of the gram-negative bacilli present in water and soil as these bacilli may survive in hostile conditions.^[2]^

In this study, we reported a case of community-acquired pneumonia with thoracic empyema, and sepsis caused by *B pseudomallei* and identified this case as the first case of melioidosis in Mato Grosso do Sul, Midwest state of Brazil.

## 2 Case report

A 42-year-old man who lived in a rural area worked as a bricklayer 3 days prior to the onset of disease symptoms. He was admitted into the emergency department, on 04/04/2016 with a medical history of non-productive cough, dyspnea, myalgia, diffuse abdominal pain, and enterorrhagia for 10 days. He was a smoker and alcoholic patient. The physical examination indicated the conditions including a toxemic appearance, emaciation, consciousness, disoriented behavior, icterus (3+/4+), dehydration, and fever. His respiratory rate and cardiac frequency was 40 breaths/min and 140 beats/min, respectively. Pulmonary auscultation revealed a vesicular murmur, snoring sounds, and the presence of basal crackling rales in the left hemithorax. The abdomen was distended and painful owing to hepatomegaly and lower limb edema was observed.

The results of laboratory examinations indicated the levels of hemoglobin (9.8 g/dL), hematocrit (26.5%), leukocyte count (39.640 per mm^3^), stab neutrophils (5%), neutrophils (84%), platelets (2.46 million per μL), creatinine (3.7 mg/dL), urea (235 mg/dL), K^+^ (3.6 mEq/L), Na^+^ (124 mEq/L), amylase (38 UI/L), lipase (238 UI/L), aspartate aminotransferase (128 U/L), alanine aminotransferase (52 U/L), total bilirubin (6.45 mg/dL), direct bilirubin (5.76 mg/dL), indirect bilirubin (0.69 mg/dL), gamma-glutamyl transpeptidase (1512 U/L), alkaline phosphatase (558 U/L), prothrombin time (1.28 INR), albumin (1.5 g/dL), and globulin 4.0 (g/dL). The arterial gasometry results indicated respiratory alkalosis with hypoxemia.

Initially, several hypotheses such as community–acquired pneumonia (CAP), abdominal sepsis, leptospirosis, secondary infections of visceral leishmaniasis, and severe dengue were proposed regarding the pathogenesis.

The results of serological tests were negative for human immunodeficiency and human T-lymphotropic viral infections, hepatitis A, B, and C, leptospirosis, visceral leishmaniasis and dengue.

The patient was admitted into intensive care units during several respiratory failures. Initially, the patient was treated using the intravenous (IV) administration of ceftriaxone (1 g) twice a day. After 48 hours, meropenem (1 g) treatment 3 times a day was initiated owing to clinical worsening. On the third day of hospitalization, *B pseudomallei* was identified in 2 blood cultures using Vitek 2 System (bioMérieux). We assessed the minimal inhibitory concentration (MIC) of specific drugs to which *B pseudomallei* was susceptible and the results were as follows: meropenem (MIC: 1), ceftazidime (MIC: 1), sulfamethoxazole (MIC: 0.5), and levofloxacin (MIC: 2).

On the sixth day after initial clinical stabilization, the patient developed a severe hypoxemia, fever (39.4°C), and hemodynamic instability. Therefore, he was subjected to endotracheal intubation.

**Figure 1:**
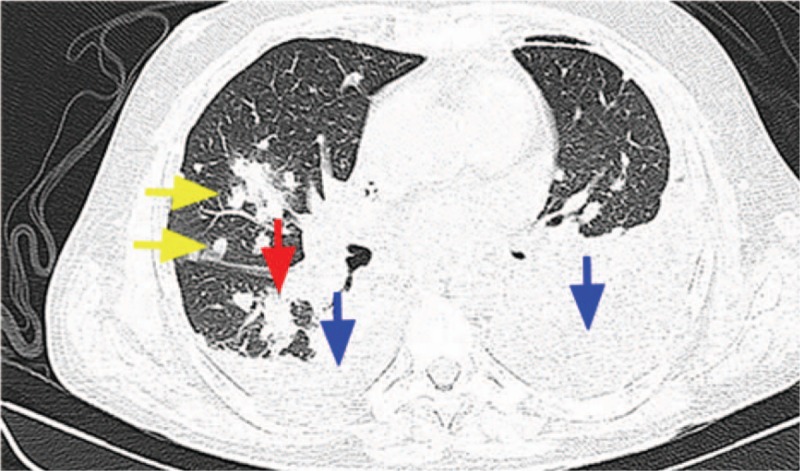
Chest computed tomography of the patient showing multiple pulmonary nodules (the yellow arrow), partial consolidation (the red arrow), and bilateral pleural effusion (the blue arrow). *Burkholderia pseudomallei* was isolated from a culture of pleural empyema, blood cultures, tracheal aspirate, and urine.

Computed tomography of the chest (Fig. 1) revealed multiple pulmonary nodules in all the lung fields. Each of these nodules was up to approximately 1.6 cm in diameter. Moreover, CCT indicated partial consolidation in the right bottom of lung, air bronchogram, bilateral pleural effusion that was moderate in the right and bulky in the left regions along with thoracic drainage. No complications were observed on the computed tomography of the abdomen.

The clinical condition of patient alternated for several times between the episodes of worsening and improvement. Therefore, administration of a combination treatment comprising trimethoprim/sulfamethoxazole (TMP-SMZ, 20 mg/kg body weight per day) and meropenem (1 g 3 times a day) stabilized the clinical condition of the patient.

We identified *B pseudomallei* using the cultures of pleural fluid, tracheal aspirate, and urine samples through Vitek 2 system. The cell isolates were subjected to 16S rRNA gene sequencing to confirm the bacterial species.^[6]^

The patient was subjected to tracheostomy and ventilatory weaning. He exhibited persistent fever for several days. As progressive clinical improvement was observed, the tracheal tube and chest drain were removed. He was referred to our outpatient clinic for treatment of the infection. TMP-SMZ was prescribed to him and he was monthly monitored up to 1 year. The antibiotic was suspended owing to the remission of disease and he is currently under annual monitoring.

Informed written consent was obtained from the patient for the purpose of publication.

## 3 Discussion

Melioidosis might present various clinical forms^[7]^ ranging from asymptomatic to severe pneumonia or sepsis similar to the case described in this study.

A total of 28 melioidosis cases were reported in Brazil until 2015.^[1]^ The majority of melioidosis cases were reported in the Northwest region of Brazil and such cases were not previously detected in the Midwest region of Brazil. Therefore, initially, the diagnostic hypothesis was associated with community diseases and endemics in our region. Additionally, the confirmation of this hypothesis was initially difficult as the symptoms of disease caused by *B pseudomallei* do not exhibit pathognomonic features.^[8]^

The incubation period of melioidosis approximately ranges from 1 to 21 days; however, disease recurrence might occur owing to persistent focus.^[9]^ Literature survey indicated that melioidosis might manifest subsequent to a latent period or early relapse in 10 to 14 days after the initiation of intravenous antibiotic therapy.^[10]^

The melioidosis is considered as an opportunistic infection as 80% of the patients exhibited one or more risk factors such as diabetes mellitus, chronic obstructive pulmonary disease, and alcoholism^[9]^ similar to the alcoholic patient in our case report.

The patient had several respiratory complications and the lungs are common organs involved in disseminated infections. Respiratory complication is associated with a severe form of infection, presenting a higher risk of mortality compared to the involvement of other organs or systems. Empyema, consolidation and nodules are common features of lung and pleural melioidosis,^[11]^ as described in this case.

Conventionally, a definitive diagnosis was difficult. The proper identification of *B pseudomallei* cultures might be ambiguous and depends on the expertise of microbiologist.^[12]^ In our study, we confirmed the bacterial species identification by performing sequencing using the polymerase chain reaction of 16S rRNA owing to the high accuracy and specificity of this method.^[13]^

Adequate antimicrobial therapy with ceftazidime, meropenem, or imipenem (IV administration) is required for 10 to 14 days, followed by 3 to 6 months of oral therapy with TMP-SMZ.^[2,14]^ In this case report, the patient exhibited the clinical worsening of symptoms while undergoing meropenem treatment. Therefore, a combination therapy of meropenem and TMP-SMZ was administered.

The mortality rate caused by melioidosis ranges from 14% to 40% among which >80% of the cases are untreated patients.^[9,12,15]^ Therefore, in our study, the satisfactory recovery of the patient reinforces the importance of the early introduction of adequate therapy to reduce mortality.

The main limitation of our study is that we were unable to define the probable source of the *B pseudomallei*, requiring expertise team to investigate the patient's environment.

This report raises doubts regarding the actual prevalence of melioidosis infection in the Brazilian population. The medical professionals must be aware of the differential diagnosis of melioidosis in community infections owing to the absence of specific symptomatic characteristics of this severe infection.

## 4 Conclusion

In this study, we described the first case of community-acquired pneumonia with sepsis caused by *B pseudomallei* in Mato Grosso do Sul state, Midwest region of Brazil and the early microbiological identification associated in adequate treatment improved patient prognostic.

## Author contributions

**Conceptualization:** Cláudia Elizabeth Volpe Chaves, Ana Cláudia Souza Rodrigues, Mara Luci Gonçalves Galiz Lacerda, Suse Barbosa Castilho.

**Data curation:** Cláudia Elizabeth Volpe Chaves, Ana Cláudia Souza Rodrigues, Mara Luci Gonçalves Galiz Lacerda, Suse Barbosa Castilho.

**Formal analysis:** Cláudia Elizabeth Volpe Chaves, Ana Cláudia Souza Rodrigues, Mara Luci Gonçalves Galiz Lacerda, Caroline Tieppo Flores de Oliveira, Ivson Cassiano de Oliveira Santos, Ana Paula D’alincourt Carvalho Assef.

**Investigation:** Cláudia Elizabeth Volpe Chaves, Ana Cláudia Souza Rodrigues, Mara Luci Gonçalves Galiz Lacerda, Caroline Tieppo Flores de Oliveira, Ivson Cassiano de Oliveira Santos, Ana Paula D’alincourt Carvalho Assef.

**Methodology:** Cláudia Elizabeth Volpe Chaves, Ana Cláudia Souza Rodrigues, Mara Luci Gonçalves Galiz Lacerda, Caroline Tieppo Flores de Oliveira, Ivson Cassiano de Oliveira Santos, Ana Paula D’alincourt Carvalho Assef.

**Project administration:** Cláudia Elizabeth Volpe Chaves, Ana Cláudia Souza Rodrigues.

**Supervision:** Ana Paula D’alincourt Carvalho Assef, Sandra Maria do Valle Leone de Oliveira, Anamaria Mello Miranda Paniago.

**Validation:** Sandra Maria do Valle Leone de Oliveira, Leonardo Roever, Anamaria Mello Miranda Paniago.

**Visualization:** Cláudia Elizabeth Volpe Chaves, Ana Cláudia Souza Rodrigues, Mara Luci Gonçalves Galiz Lacerda, Caroline Franciscato, Sandra Maria do Valle Leone de Oliveira, Leonardo Roever, Anamaria Mello Miranda Paniago.

**Writing – original draft:** Cláudia Elizabeth Volpe Chaves, Ana Cláudia Souza Rodrigues, Mara Luci Gonçalves Galiz Lacerda, Suse Barbosa Castilho, Caroline Franciscato.

**Writing – review & editing:** Cláudia Elizabeth Volpe Chaves, Ana Cláudia Souza Rodrigues, Sandra Maria do Valle Leone de Oliveira, Leonardo Roever, Anamaria Mello Miranda Paniago.

Cláudia Elizabeth Volpe Chaves orcid: 0000-0002-3004-2039.
